# Partitioning of Water Between Differently Sized Shrubs and Potential Groundwater Recharge in a Semiarid Savanna in Namibia

**DOI:** 10.3389/fpls.2019.01411

**Published:** 2019-11-13

**Authors:** Katja Geißler, Jessica Heblack, Shoopala Uugulu, Heike Wanke, Niels Blaum

**Affiliations:** ^1^Plant Ecology and Nature Conservation, University of Potsdam, Potsdam, Germany; ^2^Geology Department, University of Namibia, Windhoek, Namibia; ^3^Department of Geography and Environmental Management, University of the West of England, Bristol, United Kingdom

**Keywords:** bush encroachment, groundwater recharge, rooting depth, Savannas, stable isotopes, shrub size, *Acacia mellifera*, rain event depth

## Abstract

**Introduction:** Many semiarid regions around the world are presently experiencing significant changes in both climatic conditions and vegetation. This includes a disturbed coexistence between grasses and bushes also known as bush encroachment, and altered precipitation patterns with larger rain events. Fewer, more intense precipitation events might promote groundwater recharge, but depending on the structure of the vegetation also encourage further woody encroachment.

**Materials and Methods:** In this study, we investigated how patterns and sources of water uptake of *Acacia mellifera* (blackthorn), an important encroaching woody plant in southern African savannas, are associated with the intensity of rain events and the size of individual shrubs. The study was conducted at a commercial cattle farm in the semiarid Kalahari in Namibia (MAP 250 mm/a). We used soil moisture dynamics in different depths and natural stable isotopes as markers of water sources. Xylem water of fifteen differently sized individuals during eight rain events was extracted using a Scholander pressure bomb.

**Results and Discussion:** Results suggest the main rooting activity zone of *A. mellifera* in 50 and 75 cm soil depth but a reasonable water uptake from 10 and 25 cm. Any apparent uptake pattern seems to be driven by water availability, not time in the season. Bushes prefer the deeper soil layers after heavier rain events, indicating some evidence for the classical Walter’s two-layer hypothesis. However, rain events up to a threshold of 6 mm/day cause shallower depths of use and suggest several phases of intense competition with perennial grasses. The temporal uptake pattern does not depend on shrub size, suggesting a fast upwards water flow inside. δ^2^H and δ^18^O values in xylem water indicate that larger shrubs rely less on upper and very deep soil water than smaller shrubs. It supports the hypothesis that in environments where soil moisture is highly variable in the upper soil layers, the early investment in a deep tap-root to exploit deeper, more reliable water sources could reduce the probability of mortality during the establishment phase. Nevertheless, independent of size and time in the season, bushes do not compete with potential groundwater recharge. In a savanna encroached by *A. mellifera*, groundwater will most likely be affected indirectly.

## Introduction

Groundwater recharge, the flux of water across the water table, is arguably the most difficult component of the hydrologic cycle to measure. In arid and semiarid regions, the problem is exacerbated by extremely small recharge fluxes that are highly variable in space and time (Phillips, 1994; Stephens, 1994; Izbicki et al., 2000). Fluctuations of precipitation and often unknown effects of vegetation are additional aggravating factors (Acharya et al., 2017; Evaristo and McDonell, 2017).

In semiarid systems, mainly large but rare rain events with deep percolation result in groundwater recharge (Le Maitre et al., 1999; Scanlon et al., 2006; Moore et al., 2012). Heavy precipitation may infiltrate where it hits the ground and moves vertically to the water table, yielding diffuse recharge but at very long time scales. Vegetation affects aquifers on very short time scales by either directly extracting groundwater from saturated strata or via uptake and interception of rainwater, thereby reducing the proportion of rainfall that is eventually recharged (Le Maitre et al., 1999). The deep-rooted soil-plant system of drylands is known to be highly rain-use efficient (Schenk and Jackson, 2002; Seyfried et al., 2005).

In many semiarid regions around the world, both, climatic conditions and vegetation are currently subject to significant changes including altered precipitation patterns with more heavy rain events and changes from open vegetation into bush and thicket (Weltzin et al., 2003; Reynolds et al., 2007; IPCC, 2014). Although such shifts in precipitation regimes may have a direct positive effect on ecosystem services such as groundwater recharge, corresponding effects on plants and vegetation dynamics might be equally substantial. A number of recent studies have shown that there are differing responses of plants to rain events of different intensity (e.g. Schwinning and Sala, 2004). Dryland systems are known to be driven by highly episodic events, which is in particular true for recruitment of bush encroaching plants (Joubert et al., 2008; Joubert et al., 2013). There is limited empirical information on what effect rain intensity may have on dryland systems with already changed vegetation pattern comprising mostly adult shrubs. Lohmann et al. (2012) demonstrated in their modelling study that more intense rain events increased the individual growth of both perennial grasses and encroaching shrubs.

In savannas of southern Africa, the area-wide bush encroachment at the cost of perennial grasses is considered a rangeland problem since many decades (Scholes and Walker, 1993; Eldridge et al., 2011; Grelliera et al., 2013; Russell and Ward, 2014). In Namibia, about 50% of the savanna is purportedly affected (De Klerk, 2004). This bush encroachment causes not only a significant reduction of the economic profitability and decrease in livelihoods but also leads to changes in vegetation structure and vegetation dynamics with major implications on different functions of the ecosystem (Reynolds et al., 2007; Eldridge et al., 2011; Soliveres and Eldridge, 2014). Ecological functions include biodiversity at many levels and the stable coexistence between bushes and perennial grasses (e.g., Blaum et al., 2009; Chown, 2010; Archer et al., 2017; Hering et al., 2019; Geissler et al., 2019). Water related ecohydrological functions that are affected by changes in vegetation include the modification of local climate, changed redistribution of water, and reductions of rainfall infiltration (Sharma, 1998; Bhark and Small, 2003; Maestre and Cortina, 2004; Wilcox and Thurow, 2006; Eldridge et al., 2011; Simonin et al., 2013). Some of these consequences have been experimentally verified at a few example sites in drylands (Shachak et al., 1998; Ludwig et al., 2005). However, only few studies focus on the possible relation to potential groundwater recharge and on the interrelation of these consequences to the specific age and/or size structure of the respective bush encroacher populations. For example, larger shrubs are likely to root independently of rainwater availability. They root deeper and may even reach saturated strata, directly utilizing groundwater, deep subsurface water pools or water from the soil that moves towards the groundwater. Recent research has demonstrated that large trees in arid savannas are indeed reliant on deep water (Shadwell and February, 2017). For shrubs, deeper rooting individuals might also allow a more stable coexistence with perennial grasses. In savannas, especially in dry savannas, densely rooting grasses obtain their water from the upper soil layer whereas woody plants escape from the competition and root mainly in deeper layers using less of the upper soil water (Walter, 1954; Scholes and Archer, 1997; Sankaran et al., 2005; Ward et al., 2013). At the same time, larger shrubs have more total transpirational surface, and consequently a higher demand for water (Meinzer et al., 2005) and might therefore take up rainwater more effectively than perennial grasses and smaller shrubs, even in upper soil layers. Consequently, competition for water increases, and precipitation is less likely to move to deeper soil parts, which is in turn negatively linked to the process of groundwater recharge. In many dryland systems woody plants tend to use water opportunistically to avoid or minimize seasonal water stress. They are able to switch from using available (sub-)surface water during the rainy season to water from greater depths as the main water source in the dry season (Dawson and Pate, 1996; Xu et al., 2011). Although being essential for anticipating the effects of climate change and bush encroachment for savanna ecosystem services, the flexibility of the encroaching woody vegetation to switch between different soil layers for predominant water uptake within a season and in particular in response to different rain event properties remains poorly understood.

The objectives of this study are to quantify relationships between rain event depth, shrub size, and sources of water use of *Acacia mellifera*, one of the most important bush encroacher species in Namibia. We aim to capture indication of feedbacks of bush encroachment to water related ecosystem services such as potential groundwater recharge or the further coexistence of perennial grasses with encroaching shrubs. The pattern of rainwater use is of specific interest, as rain accounts for an important amount of the water input in these semi-arid systems. Understanding the role of bush encroacher species at various ecological levels is a prerequisite for anticipating the effects of climate and global change on ecosystems and communities therein.

In particular, we address the following questions:

1. What is the proportional contribution of soil water at different depths and soil water intended for groundwater recharge in the water use of adult shrubs of *Acacia mellifera* during the course of the growing season?
2. What is the effect of rain event depth on water source partitioning of these shrubs?
3. Do adult shrubs of different size differ in their spatial and temporal water use?

## Material and Methods

### Study Area and Time

The study presents data of three growing seasons over a period of three years, from November 2015 to October 2018. The study area is situated in the Kalahari savanna rangeland, 180 km south-east of Windhoek, Namibia at farm Ebenhaezer (23°14'S, 18°23'E). Average annual precipitation is 250 mm/a (CV = 0.41) (Ministry of Agriculture, Water and Rural Development, Namibia, 1999). Most rainfall occurs from November to April during the hot summer months. Mean annual temperature is 19.5 °C with summer peaks of up to 45 °C. Soils are classified as red sand partially underlain by calcrete (Mendelsohn et al., 2002). The savanna is described as a Central Kalahari Camelthorn Savanna. Vegetation is characterized by open shrubland with trees and shrubs sparsely scattered in a grassy landscape (trees: *Acacia erioloba, A. haematoxylon*; shrubs: *Acacia mellifera, Acacia hebeclada, Grewia flava*; grasses: *Stipagrostis spp., Eragrostis spp., Aristida spp, Pogonathria fleckii* and *Schmidtia kalahariensis*) (Mendelsohn et al., 2002). The study area has been used for livestock farming since 1907 with heavy bush encroached areas around waterholes. Currently the rangeland is used for sheep, horse and cattle farming.

### Study Species and Selection of Study Plants

The blackthorn acacia *Acacia mellifera* is one of the main bush encroachment species in the southern African savanna region (De Klerk, 2004) and is therefore of major concern to a sustainable rangeland and ecosystem management. Thirty-seven individuals of *A. mellifera* were randomly selected and measured by their canopy diameter, plant height and stem width at an inter-dune area in November 2016, several days before the first rain of the season 2016/2017. A subsample of 15 individuals was eventually chosen for the main study, ensuring the broadest range of canopy diameter and shrub height. It comprised individuals with a diameter between 35 and 952 cm and a height between 21 and 451 cm. Distance between each selected individual was >30 m to avoid effects of neighbor trees. To facilitate resampling, GPS coordinates were taken and all individuals marked with tape.

### Sampling Design of Rain Water, Ground Water, Plant and Soil Water

The natural abundance of hydrogen (δ^2^H) and oxygen (δ^18^O) stable isotopes in plant xylem water as an integrated signal of its sources was utilized to answer the research question, because root water uptake is generally considered a non-fractionating process (Dawson and Ehleringer, 1991). We compared the isotopic composition of plant xylem water with the composition in potential water sources such as water of five different subsurface layers and soil water of potential groundwater recharge.

**Figure 1:**
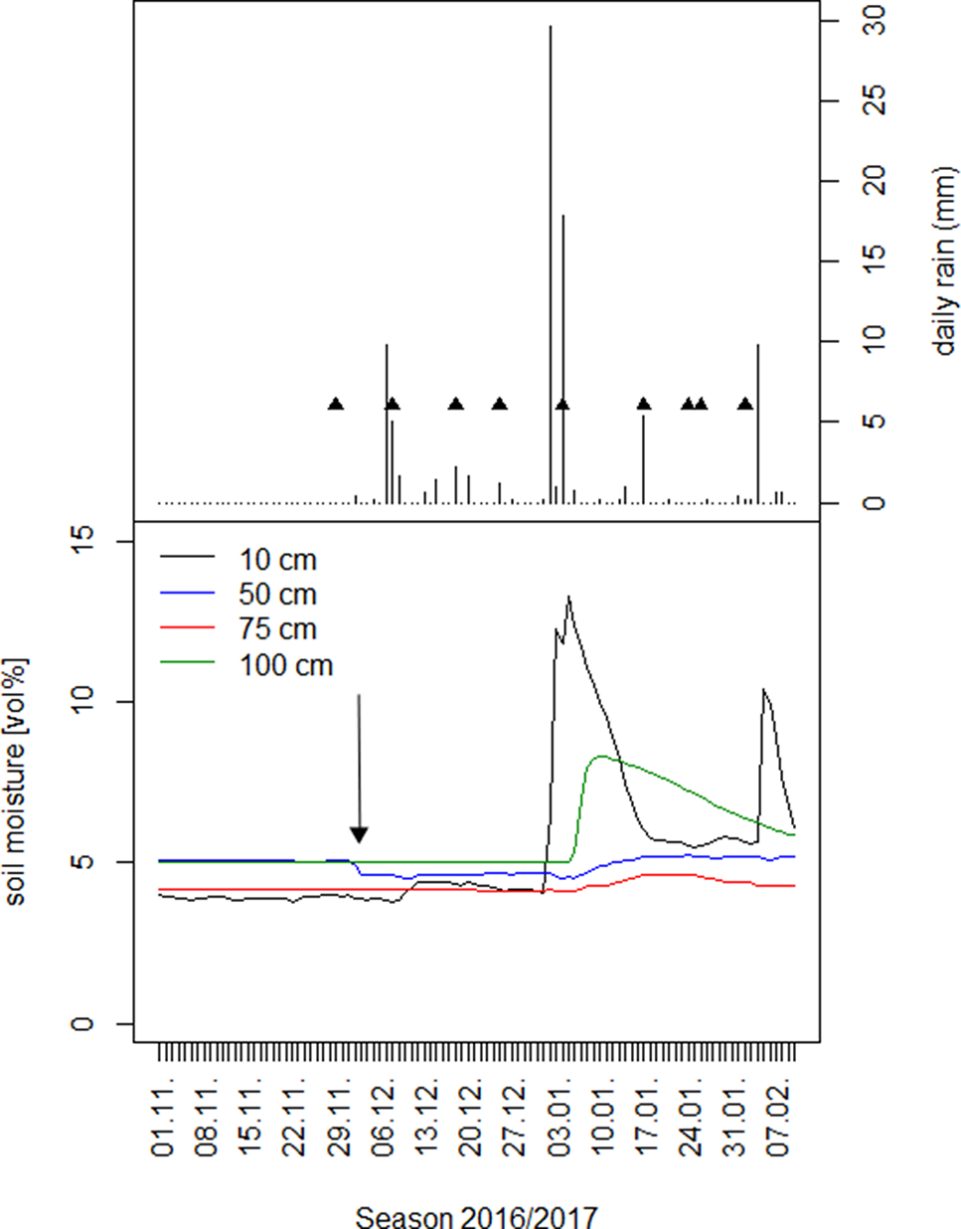
Upper panel: Date and depth of single rain events in the first half of the season 2016/2017 together with the date of the first day of xylem and water sources sampling per rain event. Lower panel: Volumetric soil moisture under *A. mellifera* shrubs in four different soil depths. The arrow is pointing to the time of first leaf flushing on 3.12.2016.

Eight rain events of more than 0.5 mm were sampled during the first half of the growing season 2016/2017 from December to February (Figure 1). These were taken from a rain gauge (covered with a funnel shaped lid to minimize evaporation) immediately after rain ceased or in the early morning when rain fell overnight. Samples representing soil water of potential groundwater recharge were collected from a nearby well (approximately 500 m) on May 26, July 17, and September 11, 2016. As the isotopic composition of deep groundwater is less affected by precipitation (Busch et al., 1992), these samples are considered representative for the entire time period. Each sample (rain or well water) was transferred into 50 ml glass bottles, the lid tightly closed, sealed with paraﬁlm, and stored frozen at -4°C until isotope analysis. Sampling of soil and xylem water was done following every precipitation event to characterize seasonal uptake patterns, with one exception. The very first sampling of xylem water occurred at the end of the dry season before the first rain and leaf flushing, on the 28th and 29th November 2016. Leaf flushing occurred on the 3^rd^ of December 2016. To characterize the temporal response of plant water-use patterns to rainfall pulses, sampling took place 1, 4, and 7 days after every rainfall event.

For each individual shrub, an approximately 10 cm long outer suberized/lignified branch was collected at the base of the live crown between 4:30 and 5:30 h in the morning. All leaves and the first centimetre from the cut side of the branch was quickly removed from the bark and green tissue below to avoid contamination with phloem sap and isotopic enrichment of water during later extraction (Stahl et al., 2013). It was immediately infolded with Teflon tape to seal it against any water loss. All prepared samples were individually packed in aluminium foil, each locked into a zipper bag and stored in a cool bag or fridge to prevent any transpiration after sunrise. We used a Scholander pressure chamber (Soil Moisture Corp., USA) for the subsequent extraction of the xylem-water. Just before extraction, the respective sample branch was recut to receive a fresh and straight cut and immediately clamped into the pressure chamber with the prepared side of the branch facing outside. A long, cut pipette tip was tightly put over the cut end to prevent any evaporation during extraction and to also prevent loss upcoming xylem-water. The pressure inside the chamber was increased continually until a steady flow of xylem-water out of the branch was achieved. This water, approximately 0.5 to 1 ml, was immediately collected in a 2-ml tube using a small pipette. The tube was tightly closed with a screw-cap and paraﬁlm, labelled and stored frozen until isotope analysis in the laboratory. We favoured this method over the cryogenic vacuum extraction because of lower organic contamination and in addition, we found no difference in isotopic composition between scholander pressure bomb and vacuum extraction (**Supplementary Figure S1**).

At each sampling day, early in the morning to minimize isotopic enrichment of water through evaporation, we used a soil auger (3 cm diameter) to dig a 1.2 m-deep hole at bare soil near the study plants to obtain soil samples in the depths of 10, 25, 50, 75, and 100 cm. These samples were immediately covered with a plastic sheet, placed into 8-ml glass vials, stored in a fridge and used to determine soil water content and isotopic ratios within the 0–100 cm layer. For the latter, water was extracted from the soil using a cryogenic vacuum distillation line (Ehleringer et al., 2000) at university in Munich. Extractions followed the guidelines of West et al. (2006) and were run for at least 90 minutes due to the very dry sandy soil samples. To concomitantly obtain the soil water content dynamics within the 0-100 cm layer, the three microsites ‘shrub’, ‘bare soil’, and ‘grass tussock’ were observed at four different soil depths of 10, 50, 75, and 100 cm using ML3 ThetaProbe Soil Moisture Sensors (Delta-T Devices). Soil moisture was measured during the whole study period.

### Isotopic Analyses

For all isotopic analyses, we used an isotope ratio laser spectrometer (LGR DT-100 Liquid Water Stable Isotops Analyzer, Los Gatos Research Inc., California, USA) at the Isotope Laboratory of the Geology Department at University of Namibia. We used the Spectral Contamination Identifier (SCI) post-processing software to identify and quantify spectral contamination of organic compounds, and to correct the isotope values of contaminated water samples. About 0.5 µl of water was injected and the δ^18^O and δ^2^H ratios determined with a precision of ±0.8 for δ^2^H and ±0.2‰ for δ^18^O. ^18^O and ^2^H values are reported in delta notation which expresses the isotopic composition relative to a standard (Vienna Standard Mean Ocean Water, V_SMOW_) on a per mil (‰) basis: 

δ180  orδ2H[000]=(RsampleRstandard−1)×1000

where *R* *_sample_* and *R* *_standard_* are the stable isotopic ratios (^18^O/^16^O or ^2^H/^1^H) of the sample and of standard water (Standard Mean Ocean Water, V_SMOW_), respectively.

### Data Analyses

All statistical analyses were performed using R 3.2.3 (R Core Team, 2016) and the R-packages “nlme” (Pinheiro et al., 2017), “lme4” (Bates et al., 2015) “piecewiseSEM” (Lefcheck, 2015) and “simmr” (Parnell, 2016), the latter using “ggplot2” (Wickham, 2016) and the software JAGS version 4.3.0 (Plummer, 2017).

Linear-mixed effects models (lmer, R-package ‘lme4’) were used to test for fixed effects of shrub size (expressed as canopy diameter) in interaction with rain amount, day after the rain event and time within the growing season (expressed as rain event nr.) on square root transformed δ^2^H and log_e_ transformed δ^18^O of the xylem water with day after rain nested in rain event and individual shrub ID as grouping variables in the random intercept part of the model. We assessed collinearity between fixed effects prior to the model fit using variance inflation factor (VIF) values. All predictor variables are scaled between 0 and 1 accounting for different units and to facilitate comparing coefficients for different predictors within the model (Gelman and Hill, 2007). To obtain minimal adequate models for each response variable transformed δ^2^H and transformed δ^18^O of the xylem water, we simplified the maximal model by removing all nonsignificant terms (P > 0.05) by stepwise-backward selection based on likelihood ratio tests (Crawley, 2007). Main effects included in significant interactions were retained in the minimal adequate models. For model simplification, we fitted all models by maximum likelihood (ML), but, to reliably quantify random effects, we additionally fitted the minimal adequate models with restricted maximum likelihood (REML; Bolker et al., 2009).

True probability distributions for the relative contribution of potential water sources to plant xylem water *via* Bayesian inferences (R-package “simmr”; Parnell, 2016) were determined. This framework uses linear stable isotope mixing models based on a mass balance equation of δ^2^H and δ^18^O. It uses Markov Chain Monte Carlo (MCMC) model fitting to estimate parameters from observed data and user-specified prior distributions. The approach allows to incorporate uncertainty (variability in isotopic signatures) in both the sources and the water-mixture (xylem water) and the residuals. Moreover, it allows accounting for more than three sources in a two isotope system where usually no unique solution exists. This Bayesian framework can however not account for cases where the isotopic fractions in xylem water exceed the isotopic limits of sources. The model will then estimate negative fractional contributions of one or more sources. Implicit assumption is that the isotopic compositions of the water mixture lie inside a convex polygon bounded by all sources within a biplot. The simmr MCMC was run for 10,000 iterations with a burn rate of 1,000 and source increments of 1% for the iterative process. Source inputs included well water and soil water of 10, 25, 50, 75, and 100 cm (Mean ± SD). Water-mixture inputs included samples from all sampling days per rain event not distinguishing between 1,4, and 7 days after the rain event because “day after rain” was non-significant in the linear mixed effect model described above. Simmr assumes that the mixture is constructed exclusively from those sources included as model inputs and that the isotopic compositions of sources differ from each other. Because root water uptake is considered a non-fractionating process (Dawson and Ehleringer, 1991), water source discrimination factors used in the mixing models were set 0 for both δ^2^H and δ^18^O. Since water naturally meets the assumption of equal elemental concentrations (O and H) among the sources, the concentration dependencies was set as equal. To effectively use stable isotope data in mixing models, sources should be isotopically distinct. A two-way ANOVA was conducted for sources including rain-water at all rain events to explain the variance of mean stable isotope signatures. This was done for both isotopes separately. Variance partitioning was followed by pairwise comparisons of overall source means using Tukey’s HSD test. The relationship between rain amount for a typical rain event between 0 and 6 mm and the main soil depth of water uptake was analysed by linear regression.

## Results

Single rain events of <6 mm were generally not reflected in any change of soil moisture under shrubs in any soil depth (**Figure 1**, **Supplementary Figure S2**). Larger rain events of around 10 mm did hardly increase soil moisture and if so, only slightly in 10 cm soil depth (**Supplementary Figure S2**). Only if occurring within short intervals of time, these moderate rain events between 6 and 10 mm became clearly reflected in an increase in soil moisture in the upper 10 cm soil depth, but again not deeper. The first of the two heavy rain events (of >10 mm) in January and March 2017 (**Figure 1**) increased soil moisture only in 10 and 100 cm depth. The second one led to a distinct increase of soil moisture in all four soil depths, but with 10 and 100 cm exhibiting the strongest and 50 and 75 cm the weakest increase. Both rain events indicate the main rooting zone of *A. mellifera* in 50 and 75 cm soil depth.

**Figure 2:**
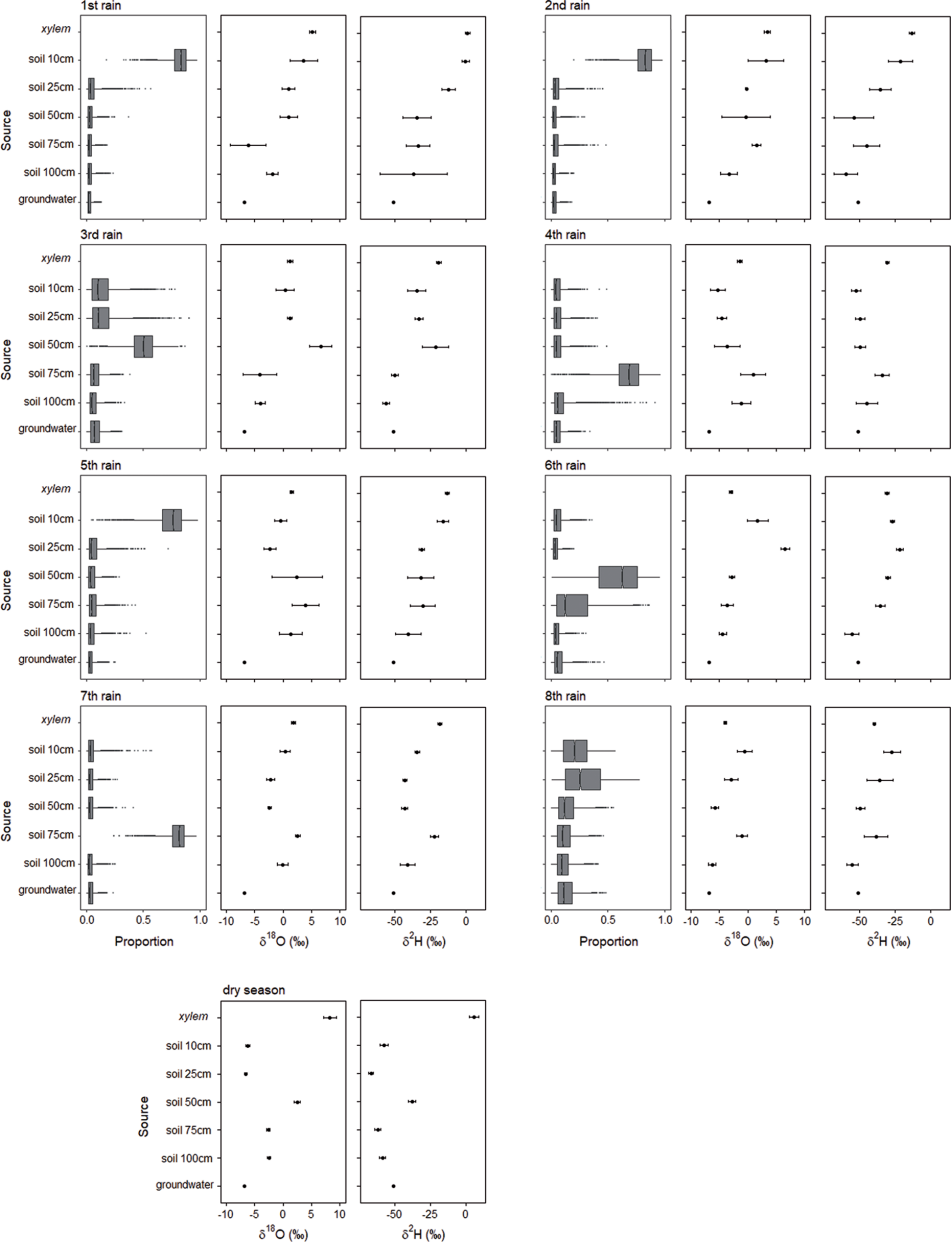
Results of simmr Bayesian mixing models showing proportional estimates (mean, 25% and 75% percentiles) of water source composition together with oxygen and hydrogen stable isotope values for *A. mellifera* before the rainy season when leaves started to flush and during 8 consecutive rain events between November and February 2016/2017 (see **Figure 1**). groundwater = soil water intended for groundwater recharge.

**Figure 3:**
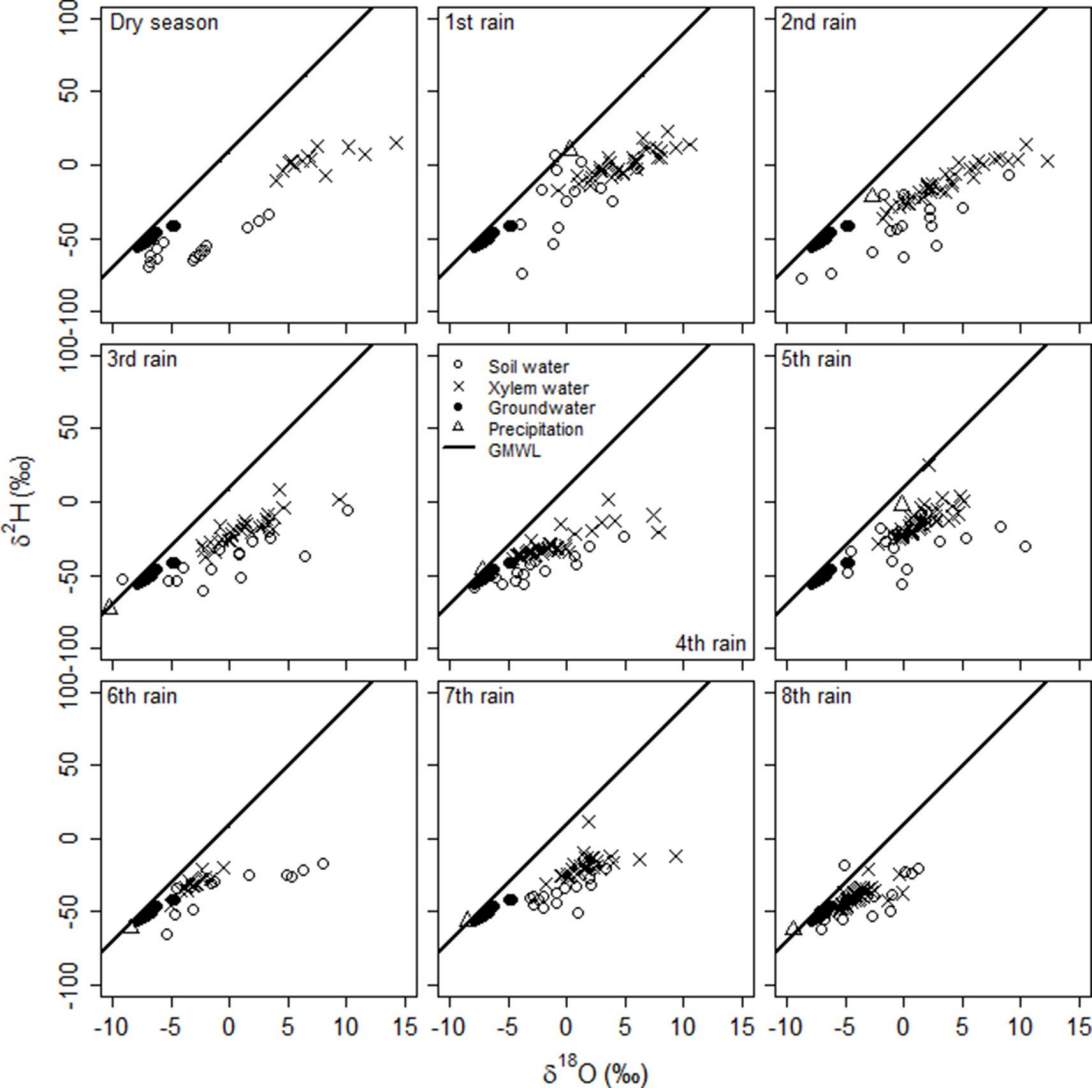
Relationships between δ^2^H and δ^18^O in the rainwater, soil water, groundwater and twig xylem water of the *A. mellifera* shrubs from November to February 2016/2017 together with the global meteoric water line (GMWL: δ^2^H = 8.17 δ^18^O + 10.35, Rozanski et al., 1993).

The overall mean δ^18^O value in soil water of all depths was higher than in rain and groundwater (**Table S1**), showing evaporative enrichment in the soil. For δ^2^H, only the mean value in the upper 10 cm of the soil profile was higher than in rain and groundwater and there was no difference between deeper soil, rain and groundwater. However, the isotopic composition of soil water at different depths changed abruptly from one sampling event to another (significant soil depth x rain event interaction: δ^18^O, F_47,76_ = 2.2, p = 0.0009; δ^2^H, F_46,75_ = 1.8, P = 0.009), reflecting a very dynamic process of soil evaporation and rainfall percolation during the study period. It also confirms our approach identifying plant source water during each rain event using separate stable isotope mixing models (**Figure 2**). All stable isotopes of soil water plot on or slightly below the GMWL (**Figure 3**) indicating soil water originates from precipitation. An evaporation trend line is observed, with the equation: δ^2^H = 3.10* δ^18^O − 36.55, r^2^ = 0.434.

**Figure 4:**
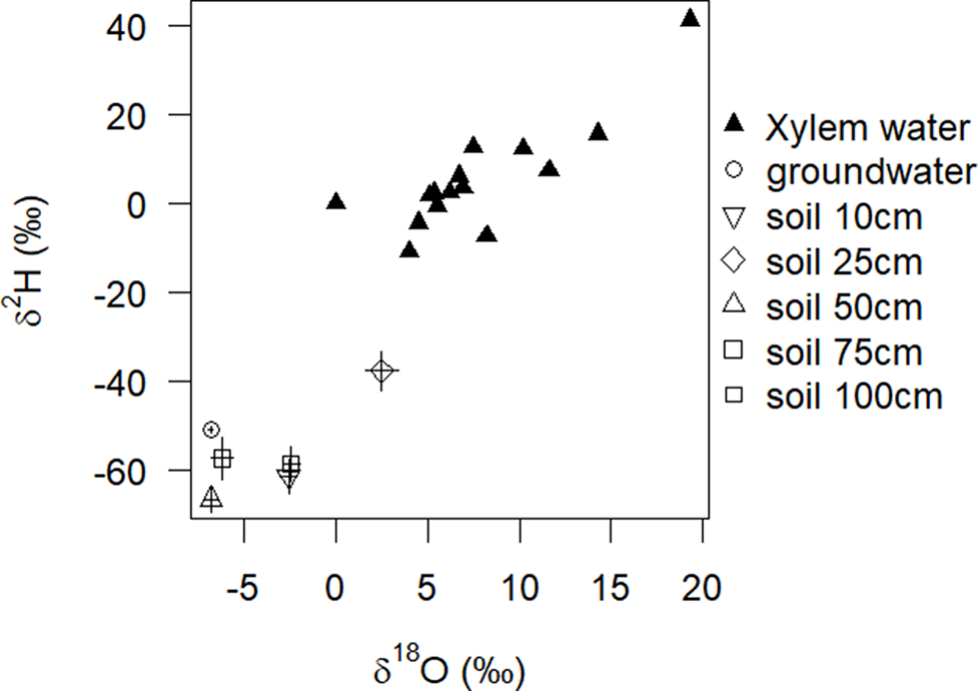
Model input for stable isotope mixing model: oxygen and hydrogen stable isotope data from different water sources at the end of the dry season used as a potential input for simmr. Solid triangles represent the stable isotope signatures of xylem water of 15 individual *A. mellifera* shrubs (n = 1). Other symbols represent, respectively, the mean and SE of isotope signatures of water sources (n = 3).

In the dry season 2016, before the first rain commenced it was only soil moisture in 50 cm soil depth, which apparently decreased at that time (Nov/Dec), when leaves started to flush (**Figure 1**).We were, however, unable to assess the water sources through an isotopic mixing model, because neither isotopic signature (xylem δ^2^H; δ^18^O) preserved an important assumption implicit in the mixing model method that the isotopic signature must be within a convex polygon, bounded by all sources within a biplot (**Figure 4**). In the other two dry seasons 2015 and 2017 in this study period, there was little if any decrease in soil moisture in 50 cm soil depth (**Supplementary Figure S2**: Nov/Dec, open 50 cm vs. bush). In 2017, the rainy season already started mid-October.

During the very first rain event of the dry season 2016 in November 2016, which was a heavy rain event, the surface soil water in 10 cm depth accounted for 75% of the twig xylem water (**Figure 2**). During the only heavy rain event, which was also part of the isotopic sampling scheme but occurred much later in the season in January 2017, soil water at 75 cm depth constitutes the main water source with 75% (**Figure 2**). In general, the use of soil water intended for groundwater recharge was subordinated. Altogether, no clear temporal pattern in using different water sources during the course of the rainy season could be established.

**Figure 5:**
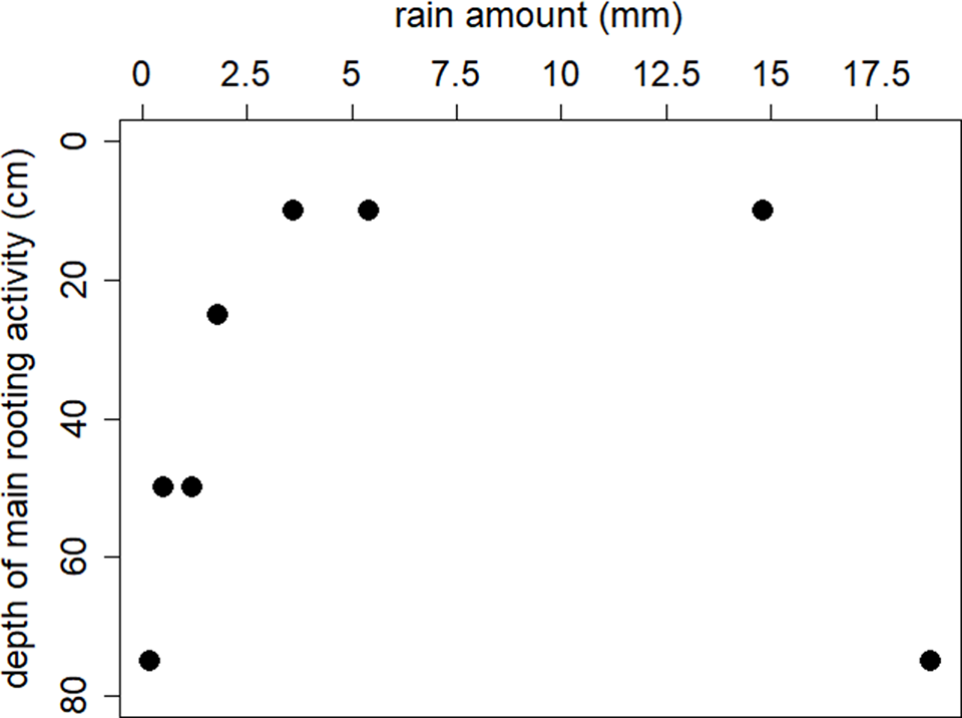
The relationship between rain amount and the main soil depth of water uptake of by adult individuals of *A. mellifera* revealed from isotopic mixing modelling.

The relationship between rain amount for a typical rain event between 0 and 6 mm and the main soil depth of water uptake by *A. mellifera* is significant (F_1,4_ = 14.0, P = 0.02), i.e., increasing rain amount up to a threshold of 6 mm/day increased rooting activity towards the upper soil layers (**Figure 5**). Heavy rain events (>12 mm/day) seemed to change main rooting activity back to lower soil layers. However, the isotopic dataset comprised only two such heavy rain events, making it difficult to draw conclusions about the real mechanisms.

**Figure 6:**
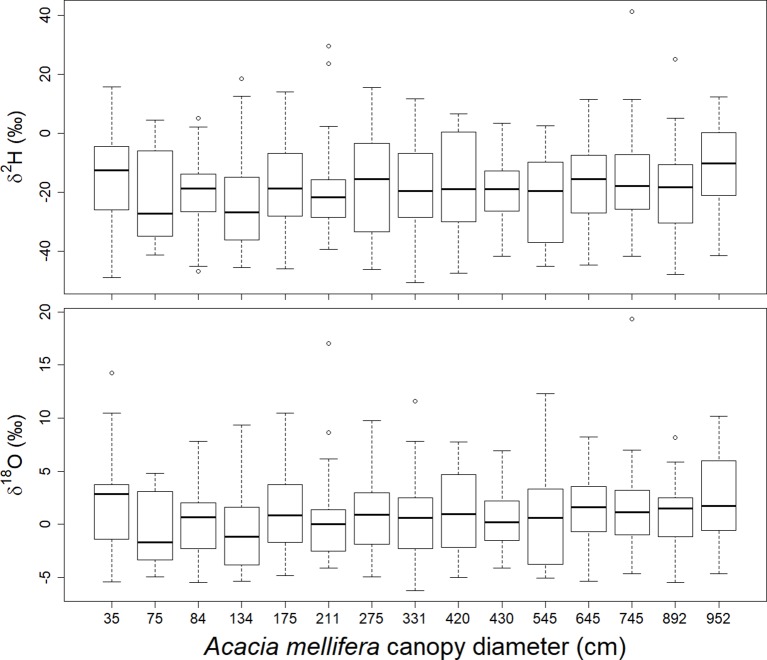
Relationship between canopy diameter of *A. mellifera* shrubs and δ^2^H and δ^18^O in the twig xylem water over the season 2016/2017. Each box contains results of 8 rain events and within each event 3 sampling days, respectively (n = 24).

Shrub size, expressed as canopy diameter, and time in the season, considered as rain event, both have a significant effect on stable isotope signatures in xylem (**Table 1**). Shrub size has a slightly increasing effect on both δ^2^H and δ^18^O ratios (**Figure 6**, **Table 1**). Only the smallest shrub size showed elevated values. Time in the season has a slightly decreasing effect on both isotopic values. Neither the parameter “day after the rain event” nor “depth of the rain event”, nor any of the interactions with shrub size had a significant effect on either isotopic ratio in xylem water.

**Table 1:** Effects of canopy size, rain event nr, rain depth, day after rain and all 2way-interactions with canopy size on both δ^2^H and δ^18^O values in the xylem.

Effect	β	χ^2^_df=1_	P	*R* ^2^_marg_	*R* ^2^_cond_
**δ** **^2^** **H**					
canopy size	**0.17**	**5.20**	**0.02**	0.05	0.73
rain event	**-0.15**	**7.30**	**0.007**	0.74	0.38
rain depth (mm)	-0.05	0.022	0.88		
day after rain	-0.15	3.65	0.06	–	–
canopy size x rain depth	–	0.013	0.91		
canopy size x day after rain	–	1.12	0.27	–	–
canopy size x rain event	–	1.47	0.23	–	–
**δ** **^18^** **O**					
canopy size	**0.15**	**6. 98**	**0.008**	0.05	0.76
rain event	**-0.12**	**6.08**	**0.014**	0.37	0.77
rain depth (mm)	-0.25	0.73	0.39		
rain depth*^quadratic^*	-1.77	1.69	0.19		
day after rain	-0.02	0.90	0.34	–	–
canopy size x rain depth	–	0.93	0.33		
canopy size x day after rain	–	0.063	0.80	–	–
canopy size x rain event	–	0.063	0.80	–	–

Shown are parameter estimates, and the approximate χ2 distribution of the test statistic, R^2^_marg_ (fixed effects alone) and R^2^_cond_ (fixed + random effects). Shrub ID and day after rain nested in rain event nr. are considered as random intercept. All response and predictor variables are scaled between 0 and 1. Significant effects are shown in bold.

## Discussion

### Proportional Contribution of Different Water Sources During the Course of the Growing Season

According to the linear stable isotope mixing model outputs soil water was in general the main source of water taken up by *A. mellifera*. Soil water of potential groundwater recharge was a subordinate source of water during the first three months of the growing season. This is consistent with a study by Kulmatiski and Beard (2013) in the Lowveld savannas of southern Krüger National Park in South Africa, where the dominating shrubs and trees seem also not to rely on soil water of potential groundwater recharge to any great extent. But it is contrary to results that have been found in other species in semiarid regions, where members of the *Combretum*, *Terminalia*,* Prosopis*, and *Acacia* genera utilize groundwater (which is most likely only soil water of potential groundwater recharge) beside soil water to support the highest transpiration demand in summer (Evaristo and McDonnell, 2017; Beyer et al., 2018). Our results instead fit with the excavation study of O'Donnell et al., (2015) in the Kalahari, where the root system of *A. mellifera* did not appear to be large enough to reach the water table or saturated zones. It means that in a woody plant encroached savanna by *A. mellifera*, groundwater will most likely be affected indirectly through the impact of these plants on the process of groundwater recharge. Soil water that reaches groundwater (or deep percolation) occurs only if precipitation water passes through the soil before the vegetation can transpire it (Wilcox, 2002; Huxman et al., 2005). Consequently, the flow is directly dependent on the overall cover of the vegetation and the extent of bare soil evaporation relative to plant transpiration. C3 woody plants such as *A. mellifera* with a relatively low ability to save water (water use efficiency) should be able to transpire large amounts of water per day and therefore be very effective in preventing any groundwater flow or deep percolation (Larcher, 2003). In southern Australia, where winter precipitation exceeds evaporation, removal of Eucalyptus scrub has led instantly to rising groundwater tables (Walker et al., 1993). Unfortunately, the depth to groundwater table has not been monitored in our study. However, boreholes for groundwater use are drilled into great depths. In southern Africa, where precipitation mostly occurs in summer, complete removal of the bush encroacher *Colophermum mopane* revealed marked increases in soil moisture in the lower 45-90 cm soil zone, which could lead to deep percolation depending on rain events (Smit and Rethman, 2000). However, authors found that incidental water losses (predominantly interception by tree canopies) were responsible for a lowering of effective rainfall and are therefore important determinants of the soil water content rather than deep soil water uptake and associated transpiration. In fact, evapotranspiration water losses from the soil even increased in plots one year after complete removal of shrubs.

Our isotopic and soil moisture data suggest that soil moisture serves as the main source of water in this sandy semiarid savanna site and accounts for over 80 % of the water needs of adult shrubs of *A. mellifera*. The global correlation between changes of soil moisture in different depths and the simple occurrence of rain events suggests a rooting activity of *A. mellifera* from 10 to 75 cm soil depth with the main activity zone in 50 and 75 cm. Isotopic analysis of xylem water during a heavy rain event suggests the main rooting activity is at 75 cm depth. The main activity zone in 50 and 75 cm fits with a recent field study along a bush cover gradient near our study site (Geissler et al., 2019). In that study, it has been found that ecophysiological traits of both shrub adults and saplings were not correlated to site specific mean soil moisture values at depths of 10 and 50 cm, while traits of annual and perennial grasses were. The proposed lower limit of active roots in 75 cm depth is in fact in the range of previously described real rooting depths for *A. mellifera* in the Kalahari (O'Donnell et al., 2015) where the authors excavated 90% of root biomass between 0 and 60 cm soil depth. However, O'Donnell et al. (2015) described the root system of *A. mellifera* as being exceptionally shallow with roots that followed an exponential distribution with depth. It suggests that in our study even the frequent missing response of soil moisture in upper soil layers to precipitation (we often observed no changes in 10 cm soil depth, although it has rained) is maybe due to woody plant water uptake from shallow soil depths and not a reflection of immediate soil evaporation or canopy interception (or both). To reduce uncertainty in reasons for soil moisture changes, we included soil moisture information of open microsites (**Supplementary Figure S1**). Results confirmed reduced movement of water into the soil due to the presence of *A. mellifera* individuals. Moreover, the isotopic composition of twig xylem water provides convincing evidence of reasonable root water uptake of the shrubs from the upper and intermediate soil layers in 10, 25, and 50 cm soil depth. Investment into shallow rooting can indeed be a successful strategy for horizontal water uptake after a low precipitation event. Nevertheless, since the main activity zone of *A. mellifera* is in 50 and 75 cm and not in the upper soil layer, and assuming O'Donnell et al. (2015) exponential root distribution with depth is prevalent, our results do not agree with the assumption that root mass distribution can serve as a proxy for rooting activity. Several authors have previously challenged this view (e.g., Eissenstat, 1992; Kulmatiski and Beard, 2013). The general inference of water uptake from measures of root biomass and changes in soil moisture might be limited because large parts of larger roots can be largely inactive. In fact, water uptake of roots preferentially goes through newly grown and relatively short-lived fine roots (Eissenstat, 1992; McCully, 1999). This is costly and the ability for a plant to survive harsh environmental conditions likely induces a cost in terms of growth capacity (Chapin, 1980), the so-called growth–stress survival trade-off. The observed increase in the 100 cm soil moisture after heavy rain events without an increase in the 50 and 75 cm soil layers might indicate a vertical subsurface flow of water, which occurs if uptake capacity of roots of *A. mellifera* has been reached. Additionally, it is likely that root channels exist. Nevertheless, the rooting activity of *A. mellifera* seems to be relatively flexible and adaptive, probably depending on the interaction between competitive situation, nutrients and rain amount, but certainly not time. Our results indicate a stationary uptake (from 50 cm soil depth) only in the beginning of the growing season, before any precipitation. However, uncertainty in the isotopic method (see **Figure 4**) prevents confidence in our conclusion. The strong deviation of isotopic composition of plant water from sources at that time point towards an isotopic fractionation either along the stem due to increasing stem photosynthesis (Dawson and Ehleringer, 1993), general limited sap flow (Martin-Gómez et al., 2016) or during root uptake that does involve transport via aquaporins or simply mycorrhization (Poca et al., 2019). Many Acacia species are known for their ability to develop micorrhizal symbiosis. Further research would be needed to obtain direct temporal quantifications of stem photosynthesis of this shrub species, root water uptake, the role of mycorrhization and related xylem water fractionation. With progressing growing season, our results on water sources of *A. mellifera* do not relate to any temporal water use pattern. In many dryland systems, woody plants tend to use water opportunistically, at switch seasonally from using water from greater depths as the main water source in the dry season to (sub-) surface water during the rainy season (Dawson and Pate, 1996; Xu et al., 2011). Gu et al. (2015) conclude that ultimately, such pattern seem to be driven by soil water availability rather than phenology, a strategy that is certainly meaningful under dryland conditions of unpredictable and erratic precipitation.

### Effect of Rain Event Depth on Water Source Partitioning of *A. mellifera*

The response to soil water availability and to rain event depth occurs on very small scale and suggested a threshold of 6 mm/day rain amount. An increasing amount up to that threshold increased rooting activity further towards upper soil layers, eventually reaching the very first centimeters of soil. It strongly suggests a regulation of rooting activity depth based on fine root growth. The regulation may be controlled by both soil moisture and soil temperature and many species show optimal soil temperatures for fine root growth (Eissenstat et al., 2013). There is a delayed activation of growth in very shallow soil layers, which get very hot compared to deeper layers and stay hot for a longer time with less rain. The changes in rooting activity depth also emphasizes that woody plant growth starts with rain amounts of less than 5 mm/day, an amount, so far, many researchers regarded to be unimportant for plant growth in savannas (Lohmann et al., 2012; Tietjen, 2016). In any case, the isotopically based results on rooting activity following small rain events justifies very shallow rooting depths for *A. mellifera.* This shrub species seems to be well adapted to utilize such small rainfall events, which occur frequently in semiarid savannas of Southern Africa (De Klerk, 2004). The results also justify that small to intermediate sized rain events must lead to increasing belowground interference competition with perennial grasses. In many semiarid systems, the main water uptake zone of perennial grasses is in these upper shallower soil layers (Schenk and Jackson, 2002; Kulmatiski and Beard, 2013; Geissler et al., 2019). The potential large overlap of rooting zones between *A. mellifera* shrubs and grasses suggests limited evidence for the two-layer hypothesis (Walter, 1954; Ward et al., 2013) that savanna woody vegetation escapes competition with grasses and primarily roots in deeper soil layers. It makes a strong argument for a more comprehensive investigation of ecohydrological traits of bush encroacher species in particular compared to non- encroacher species, including functional plasticity in root architecture and distribution. A high amount of root biomass in shallow soil can already be seen as a strategy of anticipatory competition for space with perennial grasses. In fact, this strategy and related traits may constitute the widespread success of bush encroacher species in degraded savanna rangelands. Moreover, it might form the base for a negative feedback in previously bush encroached areas towards perennial grass establishment with important effects on the reversibility of a degraded ecosystem state. The grass cover at our study site was already very low. While the rooting activity depths of adult *A. mellifera* shrubs due to small rain events suggest interference competition with shallow rooting perennial grasses, the response to larger rain events (>6 mm/day) still indicates evidence for the two-layer hypothesis (Walter, 1954). It may illustrate the base of tree/shrub-grass coexistence in the thorn bush savannas of that area and the large-scale potential for savanna restoration if shallow-rooting perennial grass cover is rehabilitated.

### Shrub Size and Water Use

A positive relation between shrub size and both δ^2^H and δ^18^O values in the xylem was observed during the first half of the rainy season, suggesting that larger *A. mellifera* shrubs rely less on upper and very deep soil water during times of seasonal rain events than smaller shrubs. This result is based on the soil depth isotopic pattern in **Figure 2** and **Table S1** and seems anomalous but is consistent with previous studies in other dryland ecosystems (Meinzer et al., 1999; Hasselquist et al., 2010; Bargués Tobella et al., 2017). It supports the hypothesis that in environments where soil moisture is highly variable in the upper soil layers, the early investment in a deep tap-root to exploit deeper, more reliable water sources could reduce the probability of mortality during the establishment phase (Ehleringer and Dawson, 1992). Additional horizontal root growth later in the ontogenesis may reflect an increasing nutrient demand associated with the maintenance of increasing canopy leaf area. The soil nutrient content is usually greatest in the upper soil layers. Furthermore, the ability of soil to support rhizobium bacteria and arbuscular mycorrhizal fungi populations, which are needed by nitrogen fixing leguminous shrubs such as *A. mellifera* is known to decrease with increased soil depth (Shukla et al., 2013). In this view, an increased competitive ability of *A. mellifera* towards shallow rooting perennial grasses may only be a secondary benefit.

### Shrub Size and Patterns of Water Uptake During the Course of a Rain Event

Although there is most likely a shrub size effect on the depth of the main rooting activity, the temporal uptake pattern within a single rain event does not depend on shrub size. Such results suggest that the upwards water flow inside the *A. mellifera* shrubs is relatively fast. Fravolini et al. (2005) observed xylem flux rates of 1 - 5 m d^-1^ in an American encroacher bush. In our study, this would result in a recovery rate of rainwater between 1 - 4.5 days for the highest *A. mellifera* individual (451 cm) and only 1.5 – 8 h for the smallest individual (35 cm).

## Data Availability Statement

The datasets generated for this study are available on request to the corresponding author.

## Author Contributions

KG, HW, and NB planned and designed the research. JH, KG, NB, and SU involved in field data collection. KG and JH analyzed the data. KG wrote the manuscript. All authors contributed to elaborating and editing the manuscript.

## Funding

This work was supported by the German Federal Ministry of Education and Research (BMBF) project OPTIMASS (01LL1302A).

## Conflict of Interest

The authors declare that the research was conducted in the absence of any commercial or financial relationships that could be construed as a potential conflict of interest.

## Supplementary Material

Supplementary Figure S1 Comparison of six plant stem water samples (*Acacia mellifera*) extracted via Scholander pressure bomb and via vacuum extraction. Left plot represents potentially contamination from Chemcorrect™ spectral analysis software indicating the relative degree of interference from contaminants in the sample. Right plot represents δ^18^O value obtained via CRDS.

Supplementary Figure S2 Upper panel: Date and depth of single rain events in the seasons 2015/2016, 2016/2017 and 2017/2018. Lower panel: Volumetric soil moisture under *A. mellifera* shrubs (bush) and open soil (open) in 2 different soil depths (10 cm dashed line, 50 cm solid line). The arrows are pointing to the time of first leaf flushing on 15.11.2015, 3.12.2016 and 17.11.2017.
