# Supplementary figures and images for: Partitioning of Water Between Differently Sized Shrubs and Potential Groundwater Recharge in a Semiarid Savanna in Namibia
Source: Front Plant Sci. 2019 Nov 13;10:1411. doi: 10.3389/fpls.2019.01411 (PMC6863959; doi:10.3389/fpls.2019.01411)

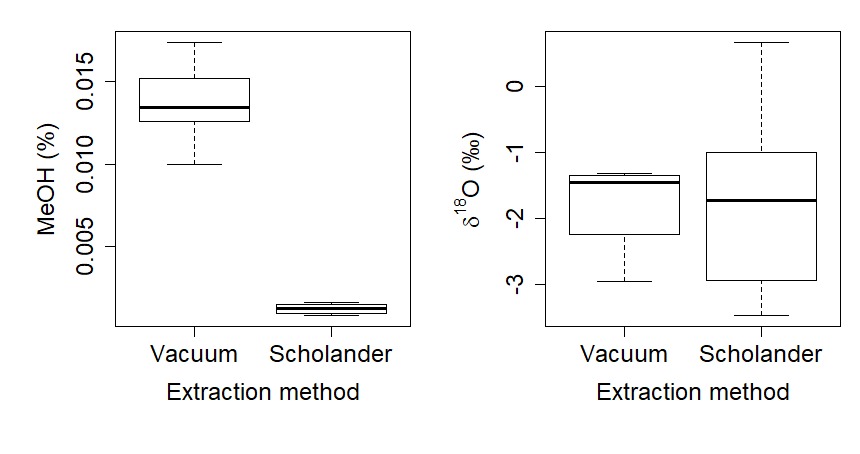

Supplement: Supplementary Figure S1 — Comparison of six plant stem water samples (Acacia mellifera) extracted via Scholander pressure bomb and via vacuum extraction. Left plot represents potentially contamination from Chemcorrect™ spectral analysis software indicating the relative degree of interference from contaminants in the sample. Right plot represents δ18O value obtained via CRDS. [file Image_1.tiff]

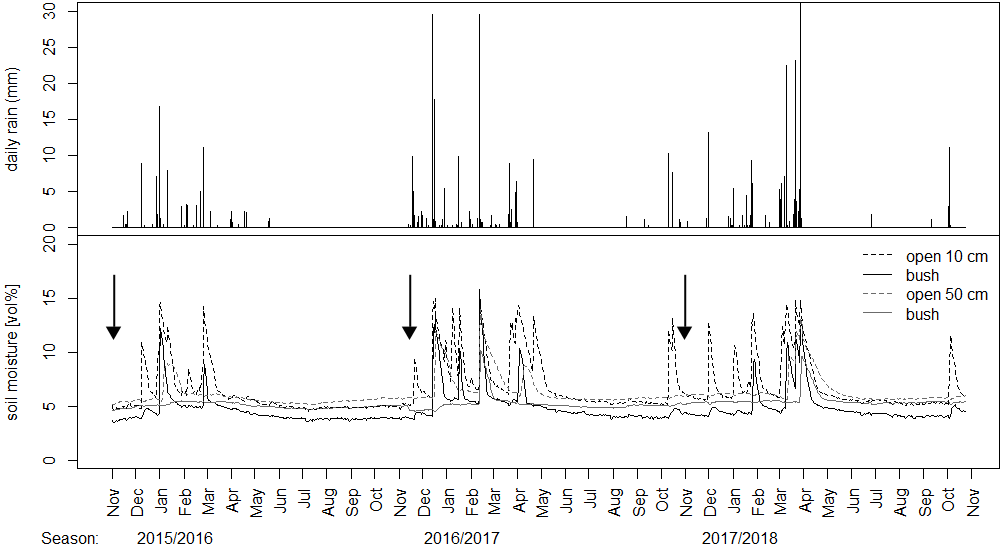

Supplement: Supplementary Figure S2 — Upper panel: Date and depth of single rain events in the seasons 2015/2016, 2016/2017 and 2017/2018. Lower panel: Volumetric soil moisture under A. mellifera shrubs (bush) and open soil (open) in 2 different soil depths (10 cm dashed line, 50 cm solid line). The arrows are pointing to the time of first leaf flushing on 15.11.2015, 3.12.2016 and 17.11.2017. [file Image_2.tiff]
