# Supplementary material for: Partitioning of Water Between Differently Sized Shrubs and Potential Groundwater Recharge in a Semiarid Savanna in Namibia
Source: Front Plant Sci. 2019 Nov 13;10:1411. doi: 10.3389/fpls.2019.01411 (PMC6863959; doi:10.3389/fpls.2019.01411)
Supplement: Supplementary file 3 [file Table_1.docx]

Table S1: Arithmetic mean (±SE) oxygen (δ^18^O) and hydrogen (δ^2^H) isotope signature of different potential water sources for *A. mellifera* water uptake during the rainy season in a savanna rangeland in semiarid Namibia and Tukey HSD Test. Each column followed by the same letter are not significantly different from another (Two-Way ANOVA; Tukey HSD test at P<0.05). Data are averaged across 8 rain events, although the interaction between rain event and Water source was also significant.

| Water source | δ^18^O (‰) | Tukey HSD | δ^2^H (‰) | Tukey HSD |
| --- | --- | --- | --- | --- |
| Rain | -5.27 ± 1.45 | A | -35.24 ± 10.25 | A,B,C |
| Soil: 0 - 10 cm | -0.71 ± 0.92 | B | -27.10 ± 6.01 | C |
| Soil: 10 – 25 cm | -0.99 ± 0.72 | B | -35.61 ± 3.75 | B,C |
| Soil: 25 – 50 cm | 0.12 ± 1.28 | B | -38.28 ± 4.44 | A,B,C |
| Soil: 50 – 75 cm | -0.08 ± 1.05 | B | -36.98 ±3.47 | A,B,C |
| Soil: 75 – 100 cm | -1.74 ± 0.63 | B | -48.11 ± 4.34 | A,B |
| Groundwater | -6.86 ± 0.13 | A | -50.86 ± 0.64 | A |
